# A High Speed Detection Platform Based on Surface-Enhanced Raman Scattering for Monitoring Antibiotic-Induced Chemical Changes in Bacteria Cell Wall

**DOI:** 10.1371/journal.pone.0005470

**Published:** 2009-05-07

**Authors:** Ting-Ting Liu, You-Hsuan Lin, Chia-Sui Hung, Tian-Jiun Liu, Yu Chen, Yung-Ching Huang, Tsung-Heng Tsai, Huai-Hsien Wang, Da-Wei Wang, Juen-Kai Wang, Yuh-Lin Wang, Chi-Hung Lin

**Affiliations:** 1 Division of Research and Development, National Yang-Ming University, Taipei, Taiwan; 2 Institute of Biophotonics, School of Biomedical Science and Engineering, National Yang-Ming University, Taipei, Taiwan; 3 Institute of Microbiology and Immunology, School of Life Science, National Yang-Ming University, Taipei, Taiwan; 4 Institute of Information Science, Academia Sinica, Taipei, Taiwan; 5 Institute of Atomic and Molecular Sciences, Academia Sinica, Taipei, Taiwan; 6 Department of Physics, National Taiwan University, Taipei, Taiwan; 7 Center for Condensed Matter Sciences, National Taiwan University, Taipei, Taiwan; 8 Taipei City Hospital, Taipei, Taiwan; 9 Taipei Veteran General Hospital, Taipei, Taiwan; University of Strathclyde, United Kingdom

## Abstract

Rapid and accurate diagnosis for pathogens and their antibiotic susceptibility is critical for controlling bacterial infections. Conventional methods for determining bacterium's sensitivity to antibiotic depend mostly on measuring the change of microbial proliferation in response to the drug. Such “biological assay” inevitably takes time, ranging from days for fast-growing bacteria to weeks for slow-growers. Here, a novel tool has been developed to detect the “chemical features” of bacterial cell wall that enables rapid identification of drug resistant bacteria within hours. The surface-enhanced Raman scattering (SERS) technique based on our newly developed SERS-active substrate was applied to assess the fine structures of the bacterial cell wall. The SERS profiles recorded by such a platform are sensitive and stable, that could readily reflect different bacterial cell walls found in Gram-positive, Gram-negative, or mycobacteria groups. Moreover, characteristic changes in SERS profile were noticed in the drug-sensitive bacteria at the early period (i.e., ∼1 hr) of antibiotic exposure, which could be used to differentiate them from the drug-resistant ones. The SERS-based diagnosis could be applied to a single bacterium. The high-speed SERS detection represents a novel approach for microbial diagnostics. The single-bacterium detection capability of SERS makes possible analyses directly on clinical specimen instead of pure cultured bacteria.

## Introduction

Conventional protocols for diagnosing bacterial infections require first to isolate a pure culture of the bacterium, followed by a determination of the identity of the isolate and an examination of the isolates responses to various antibiotics in terms of proliferation and/or viability. For such biological assays, an incubation period ranging from days to weeks or even months is required in order for the bacteria to grow to a density that can be handled by the available diagnostic tools. Over the past decade, several PCR-based methods have been developed for both the identification of bacteria [1] and the pinpoint of genes that confer antibiotic resistance [2]. Although such genotypic approaches are powerful and in most cases are able to bypass to some extent the need for bacterial culture, the assays typically require species and/or strain specific probes that may or may not be available for a particular organism. Mass spectrometry is another method that has potential as a culture-free approach for bacterial diagnostics. The proteomic information that results from the analysis of the molecular constituents of the bacteria can serve as a fingerprint that helps identify individual bacteria species and strains. However, like the PCR approach, mass spectrometry depends on the available prior knowledge on the pathogens, which may or may not exist. Lastly yet importantly, neither of the PCR or proteomics approaches can be applied to live bacteria to monitor their responses to antibiotics or to conduct functional tests.

Raman spectroscopy, providing molecular vibrational information, can become a powerful and useful method to identify molecular species. It however suffers from extremely low scattering efficiency that is approximately ten orders of magnitude smaller than that of fluorescence, obviating this technique from becoming a practical method for detecting species of low concentration. Surface-enhanced Raman scattering (SERS) has attracted a lot of attention for more than three decades [3], because it provides a means to enhance the normally weak Raman signal by several orders of magnitude. The enhancement in Raman signal is known to originate from the strong optical intensity localized within 10 nm from the surface of metallic nanostructures [4]. Using SERS, the chemical features within this range from the surface of the SERS-active substrate can be detected and analyzed in an extremely sensitive manner [5]. Efrima *et al*. first described the SERS spectrum of a bacterial cell surface [6]. Since then, several groups have reported the use of SERS-based assays for microbial detection [7], [8], [9], [10], [11], [12], [13]. Most SERS analyses however face a detrimental challenge: in that the signals captured often vary too much for practical use. These fluctuations arise mainly from a lack of homogeneity in the SERS-active substrates and due to inconsistent binding between the bacteria and the substrate. We demonstrate here a novel application of the SERS technique for assessing the biology of bacteria based on our newly developed SERS-active substrate which is made of an array of Ag-nanoparticles imbedded in anodic aluminum oxide (AAO) with nanochannels [14]. It exhibits highly reproducible Raman signal enhancement factor due to the uniform narrow gaps between Ag-nanoparticles. We have furthermore optimized the experimental protocols to promote adherence of bacteria onto the substrate. The SERS platform's sensitivity and stability is high enough to support single-bacterium detection. We report here a unique application of this SERS system to the analysis of fine changes in the bacterial cell wall during the bacterium's different growth stages and of the bacterium's response to antibiotic treatment during early period of antibiotic exposure.

## Results

### Microbial detection by SERS spectra

**Figure 1:**
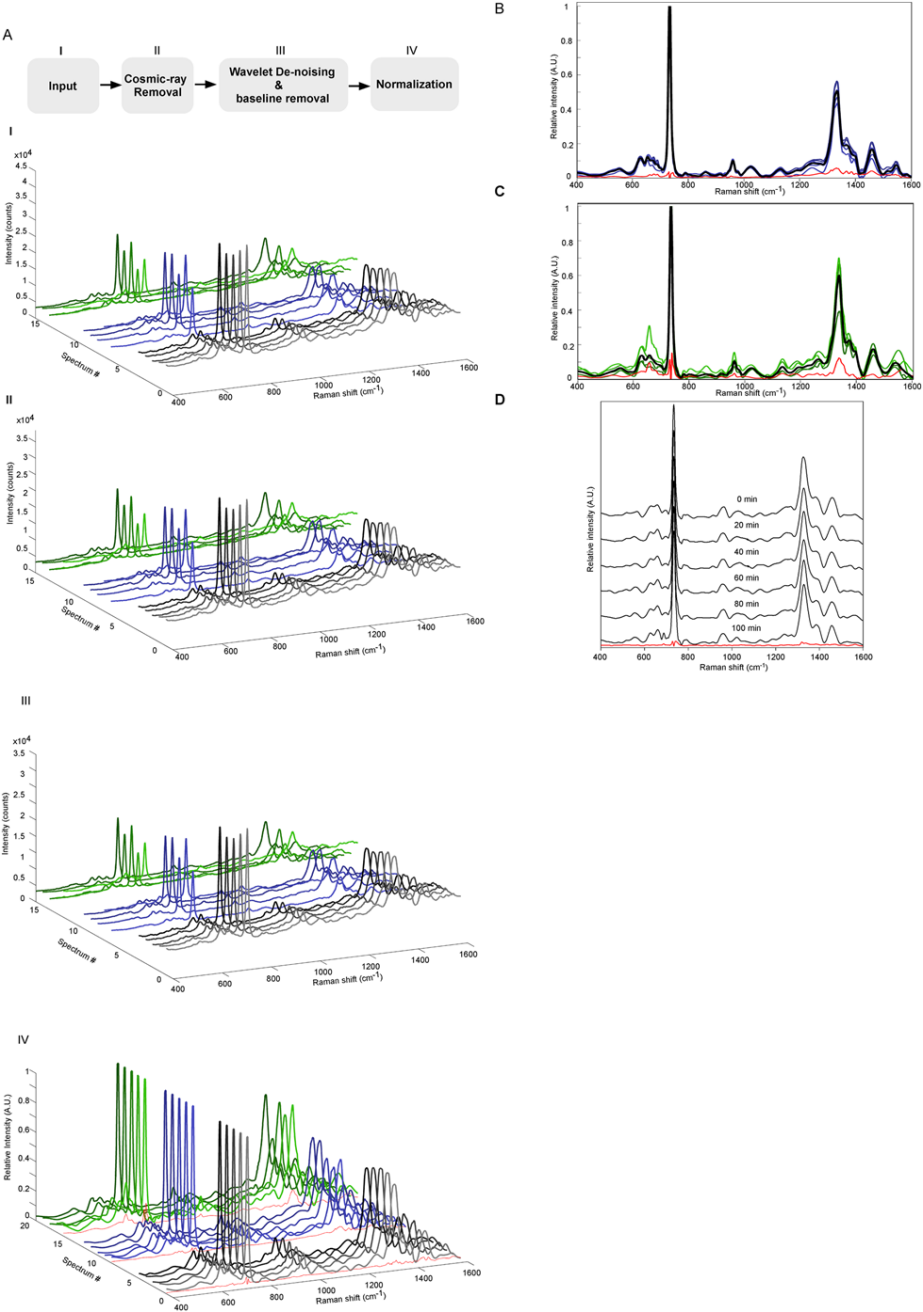
The SERS detection platform and dataset normalization. (A) Five SERS spectra were taken from the same *S. aureus* bacteria (black traces), different clusters of bacteria present on the same substrate (blue traces), or bacteria on different substrates (green traces). The raw dataset (I) were subjected to three steps (II, III, IV) of processing to normalize the spectra by the constant value of 732 cm^−1^. (B–C) Normalized SERS spectra from five data points of bacteria on the same substrate (B), or bacteria on different substrates (C) are superimposed with each other. (D) Normalized SERS spectra of the same *S. aureus* bacteria that were recorded over time as indicated. Standard deviations along the spectrum are shown in red.

**Figure 2:**
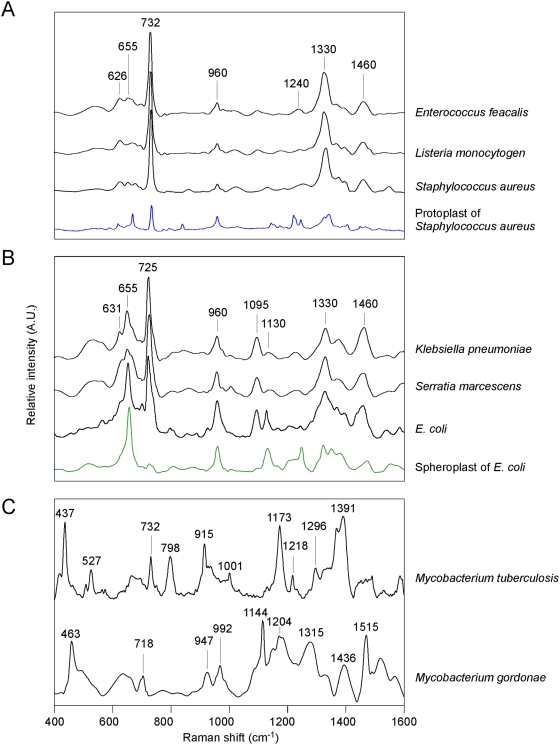
SERS spectra of bacteria with different cell wall compositions. Spectra of (A) various Gram-positive bacteria are presented as well as that of cell wall-less protoplasts of *S. aureus* (blue trace), (B) various Gram-negative bacteria are presented as indicated as well as that of cell wall-less spheroplasts of *E. coli* (green trace), and (C) two species of *Mycobacterium*. Each SERS profile stands for the mean spectrum averaged from more than 10 samples.

**Figure 3:**
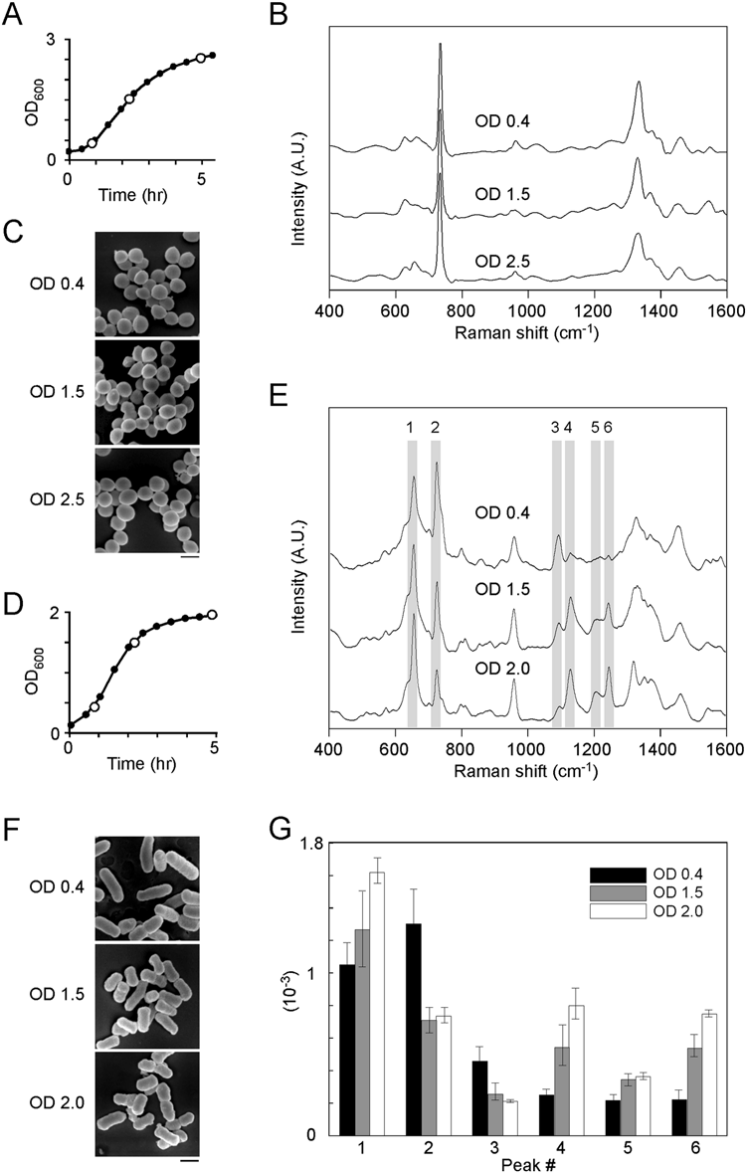
SERS spectra of *S. aureus* and *E. coli* obtained at different growth phases. (A–C) Gram-positive *S. aureus* was grown to OD_600_ 0.4, 1.5 and 2.5 and then harvested (open circles, A); the spectra (B) and SEM images (C) were then recorded. No significant changes were present. (D–G) Gram-negative *E. coli* was grown to OD_600_ 0.4, 1.5 and 2.0 and then harvested (open circles, D); the spectra (E) and SEM images (F) were then recorded. Six SERS peaks (#1∼#6) showed the progressive changes as the OD_600_ value of the culture increased. (G) Quantification (mean±S.D.) of the altered SERS peaks is shown in (E).

**Figure 4:**
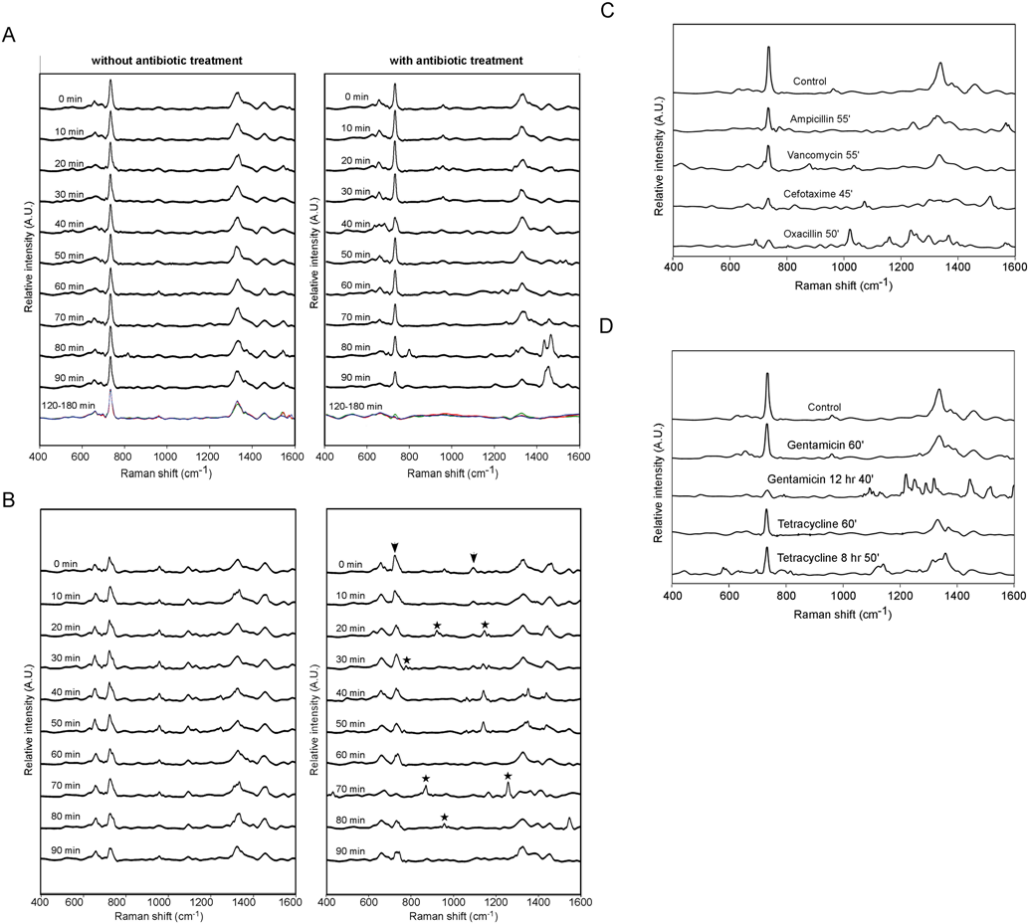
Antibiotic-induced SERS spectral changes are indicative of the bacteria's antibiotic sensitivity. (A) Sequential SERS recordings of *S. aureus* with or without the exposure to oxacillin. Time periods of drug exposure are indicated. (B) Sequential SERS recordings of *E. coli* with or without the exposure to ampicillin. Noted that the decrease in the peaks at 725 and 1095 cm^−1^ indicates an inhibition of bacterial proliferation. (C) Spectra of *S. aureus* after treated with various different antibiotics as indicated. All of the antibiotics target the bacterial cell wall. The time period shown for each antibiotic treatment corresponds to the beginning of observing significant spectral changes. (D) Spectra of *S. aureus* after treated with antibiotics that inhibited bacterial protein synthesis for time periods as indicated. A characteristic SERS response was not noted until after 9∼13 hr of treatment.

**Figure 5:**
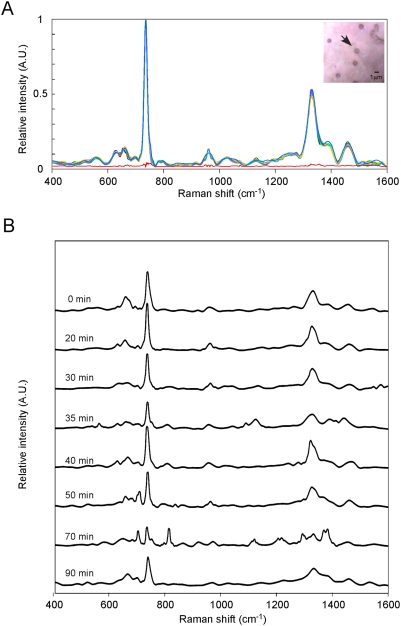
SERS-based microbial diagnostics of a single bacterium. (A) A single *S. aureus* resolved under light microscopy (arrow, inset) was subjected to SERS detection every 10 min for 90 min (various colored traces); the standard deviation among all recordings along the spectrum are shown in red. (B) Sequential SERS spectral evolution of a single live bacterium of *S. aureus* on exposure to vancomycin, which is known to actively disrupted bacterial cell wall.

Typical examples for bacteria detection using our SERS system and subsequent data processing are demonstrated in **Fig. 1**. Gram-positive bacteria *Staphylococcus aureus* (*S. aureus*) adhered to the SERS-active Ag/AAO substrate were detected using high numerical aperture (NA>0.9) objectives. Given the density of bacteria applied to the substrate and the field of microscopic detection, it is estimated that a sensible SERS readout is generated from the ensemble signal of one to seven bacteria. Acquisition of a SERS spectrum took only 1∼3 sec and this should be compared with the minutes of integration time needed using conventional Raman spectroscopy. **Fig. 1AI** shows the raw SERS readouts from the same cluster of bacteria over time (*black traces*), or from five different clusters of bacteria plated on the same Ag/AAO substrate (*blue traces*), or from bacteria on five different SERS-active substrates (*green traces*). The raw SERS datasets were then processed (through steps **II**, **III**, and **IV**) using algorithms developed in our laboratory to remove noise due to three major sources: a median filter with noise estimation was applied to eliminate any sharp variations caused by cosmic rays (**Fig. 1AII**), a wavelet de-noising technique was used to smooth out high-frequency noise, and iterative curve fitting to estimate and remove the background baseline due to the noise effect of environmental light (**Fig. 1AIII**). Finally, the spectra were normalized to an identical standard for comparison (**Fig. 1AIV**). This was done using either the constant sum of photon counts between 400 and 1600 cm^−1^ (**Fig. 4** **&** **Fig. 5B**), or the constant value of photon count of the highest peak at 732 cm^−1^ (**Fig. 1AIV, B–D** , **Fig. 2** **,** **Fig. 3B&E** **,** **Fig. 5A**). The variations among the normalized traces (SD values) are also shown (*red traces*, **Fig. 1AIV, B–D** **,** **Fig. 5A**).

The SERS spectra obtained from different clusters of bacteria on the same substrate and different bacteria on substrates of different lots are superimposed in **Fig. 1B** and **1C**, respectively. In both datasets that were normalized by 732 cm^−1^ peak, eight common peaks emerged at 626, 655, 960, 1026, 1240, 1330, 1396 and 1460 cm^−1^. Individual SERS spectra are highly similar within their own dataset, as well as when comparing with each other from the two datasets. The SERS spectrum for *S. aureus* shown here is very similar to the data previously reported by Premasiri et al [8].

In **Fig. 1D**, time-lapsed recordings of the SERS signals are shown from the same cluster of *S. aureus* over 100 min. The serial dataset was highly similar to the above datasets with less than 5% standard deviation (*red trace*).

### The characteristics of the SERS profiles reveal the chemical features of the bacterial envelope

The SERS spectrum generated by illuminating the whole bacterium as it interacts with the silver nanoparticles should reveal principally the molecular composition within ten nanometers of the outermost bacterial envelope [14]. It is well known that the components and architecture of the bacterial envelope are very different between Gram-positive and Gram-negative bacteria and that this underlies their different affinities with Gram stain. We anticipated that such structural differences should be clearly visible by SERS analysis. In **Fig. 2A–C**, each SERS spectrum representing the bacterium is a mean spectrum averaged from more than 10 samples. Note that all SERS spectra of the various Gram-positive species were similar to one another, but these were noticeably different from the Gram-negative species or the *Mycobacterium* species. Interestingly, when *S. aureus* was treated with lysostaphin and lysozyme to remove the cell wall, the resulting protoplasts exhibited a remarkably different SERS spectrum (blue trace, **Fig. 2A**) comparing to the intact bacteria; particularly, the intensity at 732 cm^−1^ was drastically decreased.

Similarly, all SERS spectra of the various Gram-negative bacteria were more related to each other than to Gram-positive bacteria and contained main peaks at 631, 655, 725, 960, 1095, 1130, 1330 and 1460 cm^−1^ (**Fig. 2B**). Treating Gram-negative *Escherichia coli* (*E. coli*) with lysozyme also generated a cell wall-less spheroplast, the SERS spectrum of which was noticeably different from the intact bacteria. Specifically, the peaks at 725 and 1095 cm^−1^ almost disappeared (green trace). If we consider the protoplast and spheroplast results together, they suggest that most of the SERS signals for the intact bacteria indeed originate from the bacterial cell wall. The SERS spectra for *Mycobacterium tuberculosis* and *Mycobacterium gordonae* (a non-tuberculosis *Mycobacterium* species or NTM), on the other hand, showed individual and distinct patterns that differed from both the Gram-positive and the Gram-negative bacteria tested in this study (**Fig. 2C**). These unique profiles might reflect the presence of mycolic acid and other unique components that are found in cell envelope of *Mycobacterium*.

### Gram-negative bacteria in different growth phases exhibit discernibly different SERS spectral profiles

The high sensitivity of the SERS detection enabled us to carry out a novel study that addressed the changes in the bacterial envelope as microbes divide at different rates. In **Fig. 3A**, the density of Gram-positive *S. aureus* in broth culture was quantified by measuring the turbidity or optical density at 600 nm (OD_600_). Three distinctive growth phases at OD_600_ 0.4, 1.5 and 2.5 were used to represent bacterial growth at the beginning of exponential phase, the middle to late exponential phase and stationary phase, respectively. It was found that the Gram-positive bacteria in these different growth phases gave rise to very similar SERS spectra (**Fig. 3B**); furthermore, they had indistinguishable morphologies under scanning electron microscopy (**Fig. 3C**).

In contrast, when a similar experimental protocol was applied to Gram-negative *E. coli*, we noticed that the SERS spectra obtained from the bacteria growing at the start of exponential phase (OD_600_ = 0.4, **Fig. 3D**), towards the end of exponential phase (OD_600_ = 1.5) and at stationary phase (OD_600_ = 2.0), showed characteristic changes in their SERS profiles (**Fig. 3E** and quantified in **Fig. 3G**). Particularly, we found that the intensity of peaks at 655 cm^−1^ (#1), 1130 cm^−1^ (#4), 1219 cm^−1^ (#5) and 1245 cm^−1^ (#6) progressively increased as the bacteria moved from exponential phase to stationary phase, while over the same period the intensities of the peaks at 725 cm^−1^ (#2) and 1095 cm^−1^ (#3) gradually decreased. In addition to the SERS changes, the SEM images revealed a decrease in the aspect ratio of the rod-like bacteria. This indicated a “shortening” of the *E. coli* cells as the bacteria growth approached a maximum (**Fig. 3F**). Therefore, subtle structural alterations in the cell morphology might contribute to the SERS changes observed.

### Early SERS spectral changes are indicative of bacteria's susceptibility to antibiotic treatment

Taking advantage of the highly sensitive SERS detection, we next addressed if the SERS spectra could be used for assessing bacteria's susceptibility to antibiotics, especially the beta-lactam antibiotics, the main action of which centers on disrupting the integrity of the bacterial envelope. In **Fig. 4A**, time-lapsed SERS recordings were conducted on the same cluster of oxacillin-sensitive *S. aureus* that were treated with the antibiotic (*right panel*) or control solution (*left panel*). The recordings started before addition of the antibiotic (0 min) and were then collected every 5 min after addition of the antibiotic at 5 mg/L or five-fold above the known minimal inhibitory concentrations (MIC). Without the antibiotic, there is no obvious change in the SERS profile (*left panel*, **Fig. 4A**). In the presence of the antibiotic, we noticed the appearance of new SERS peaks at 50 min and these in conjunction with a great decline in the main peak at 732 cm^−1^. Interestingly, the newly emerged peaks then rapidly disappeared over the next 10 to 20 min and the 732 cm^−1^ peak recovered with the spectrum appearing to return to a state very similar to the control profile. Typically after more than two hours of antibiotic treatment, the cell wall disruption prevailed and the change of SERS profile became irreversible (*the color traces in the bottom*, *right panel*, **Fig. 4A**).

Similarly, when an ampicillin-sensitive *E. coli* was treated with 20 mg/L of ampicillin or five-fold above the known MIC, there were obvious changes in SERS spectra at about 20 min after the antibiotic exposure (**Fig. 4B**). Unlike in Gram-positive bacteria where the early SERS perturbations caused by oxacillin (**Fig. 4A**) showed a transient recovery, the SERS changes detected in the Gram-negative *E. coli* following ampicillin treatment were continuous and progressive, characterized by the appearance (asterisks) of new SERS peaks over the 90 min recording period. Notably, the declines in the SERS peaks at 725 cm^−1^ and 1095 cm^−1^ (peaks #2&3, **Fig. 3G**) in the early phase of the ampicillin treatment seem to be indicative of a slowing in Gram-negative bacterial growth (arrows, **Fig. 4B**). These results support the notion that one of the immediate early effects of antibiotic treatment is an inhibition of microbial proliferations and this precedes the death of the bacteria.

In **Fig. 4C**, we found that *S. aureus*, when treated with other antibiotics that target the cell-wall such as ampicillin, vancomycin and cefotaxime, showed characteristic SERS spectral changes within an hour for all the antibiotics used. On the other hand, when *S. aureus* was treated with gentamicin or tetracycline, which inhibit protein synthesis, there were no significant SERS spectral alterations at the early-stage treatment; only until after a relatively long treatment (9∼12 hr) were we able to observe discernible SERS changes (**Fig. 4D**).

### The SERS analyses can be applied to a single bacterium

To test the sensitivity of our detection platform, we have conducted experiments on a single bacterium. As exemplified by **Fig. 5**, we were able to distinguish individual *S. aureus* bacteria shown to be adhered to substrate under the microscopy; a SERS signal was directly acquired from one of them at a time (arrow, **Fig. 5A**, inset). The time-lapsed SERS recording from such a single bacterium was extremely stable over time (**Fig. 5A**). Expanding this, it was found that the SERS-based antibiotic sensitivity test could also be applied to examine a single bacterium. As shown in **Fig. 5B**, on adding vancomycin to a single antibiotic-sensitive *S. aureus*, it was possible to obtain the characteristic SERS spectra (**Fig. 5B**) indicative of this bacterium's susceptibility to the antibiotic.

## Discussion

We report here a new diagnostic platform that reveals chemical features associated with the bacterial envelope. Using the cell wall SERS spectra as fingerprints for individual bacteria, this system can potentially differentiate known or even unknown microbes. However, although some bacteria, such as *S. aureus* and *E. coli*, do possess hallmark SERS spectra for species identification, there are bacteria whose SERS profiles significantly overlap such that this hinders differential diagnosis. Furthermore, at the subspecies level, although certain strains of *Klebsiella pneumoniae* are distinguishable by SERS due mainly to variation in capsule formation (data not shown), the current SERS detection method alone cannot unequivocally tell, within this bacterial species, one strain from another. Further improvements in data analysis might enhance the differentiation power; however, we do realize that compared to genome sequencing, PCR reactions or even mass spectrometry-based proteomics analysis, the SERS spectra described here lack the specificity at a molecular level that will allow the definitive assigning of bacterial identity. Nevertheless, taking advantages of the new method's convenience, rapidity, stability and especially its high sensitivity, we demonstrate here several novel applications and functional tests that cannot be easily achieved by other platforms. Specifically, we found that the microbes' proliferation state and its susceptibility to antibiotics can be rapidly uncovered by studying the dynamic changes that occur in the SERS profiles in live bacteria. Since SERS detection is based on revealing the “chemical features” of the bacterial envelope rather than by monitoring the progress of a biological event, such as division, the SERS method is especially useful for the analysis of slow-growing bacteria, which typically may take weeks during laboratory tests.

One novel use of SERS described in this study is the analysis of the fine structural changes in the bacterial cell wall during the various stages of bacterial growth (**Fig. 3**). Published research in this area is limited and sometimes controversial. For example, a series of biochemical studies have shown that the relative amounts of individual components of the bacterial envelope do indeed vary during the bacterial life cycle as they are subjected to different degrees of biosynthesis and degradation [15], [16]. Such changes in the macromolecular composition on the cell wall have been suggest as associated with alterations in inter-bacterial adhesion as *E. coli* K-12 progresses through the division cycle [17]. In contrast, experiments using infrared spectroscopy have not reveal any significant changes in the cell wall composition of *Proteus stutzeri* comparing actively dividing cells with those that were not [18]. Using SERS, Premasiri et al. also reported that were only minimal differences in the bacterial cell wall over the life cycle of *Bacillus anthracis* [19]. We found in this study that although the SERS spectra of Gram-positive *S. aureus* remains relatively constant at different levels of proliferation, the SERS spectra of Gram-negative *E. coli* exhibited a number of characteristic changes as the rate of microbial division decreased. It is well known that some biogenesis processes in bacteria are not coupled exactly to each round of cell division. For example, completion of the replication of the 5 million base-pairs genome of *E. coli* may take more than 40 min even while the bacteria continue to divide once every 20 min. We reason that for the Gram-negative bacteria, some of cell wall biosynthesis and maturation may still be underway even after cell division and that this may account for the SERS spectral changes when the Gram-negative bacteria move from exponential phase toward stationary phase (**Fig. 3D–G**).

In view of the chemical heterogeneities and structural features in bacteria, several important points call for attention in the discussion of the molecular interpretation of the SERS profiles presented in this study. First, it has been known that the SERS profile reflects the molecular compositions that are in close proximity with the SERS substrate, indicating that what we obtained in the spectra of bacteria should merely reflect the composition of cell wall. Second, the aromatic groups in the cell wall compositions usually have relatively strong affinities toward silver nanoparticles in our SERS substrates, and are expected to be more probable candidates for SERS activity [20]. Third, the flexible structural surface appendages (such as fragellae, fimbriae, fibrils, etc.) and the tenuous capsule around bacteria, which contains polysaccharides, glycoproteins, lypopolysaccharides and uronic acids, on top of the bacterial outer surface may not prevent the cell wall structure from exposing to the enhanced localized field surrounding the Ag nanoparticle surface. The spectral repeatability shown here further demonstrates that these substances may not play any significant role in the observed SERS spectra. Fourth, as the cell wall is made of many components with similar molecular compositions, it is very likely that these components all share similar Raman signatures. The most prominent features in the SERS spectra of Gram-positive and Gram-negative bacteria (**Fig. 2A and B**) are located at about 730 and 1330 cm^−1^ which were assigned to the purine ring breathing mode and the C–N stretching mode of the adenine part of the lipid layer components of the cell wall [7]. Jarvis, et al, on the other hand, attributed the 730-cm^−1^ peak to a glycosidic ring mode from the cell wall peptidoglycan building blocks, N-acetyl-d-glucosamine (NAG) and N-acetylmuramic acid (NAM) [10]. In our study, the 730-cm^−1^ peak is greatly reduced in both of the protoplast of *S. aureus* and the spheroplast of *E. coli*, supporting that this peak is indeed mainly contributed by the cell wall, instead of purely the lipid layer. In contrast, the 1330-cm^−1^ peak in the protoplast of *S. aureus* is much smaller than that of native *S. aureus*, while that in the spheroplast of *E. coli* is comparable with that in native *E. coli*, indicating that this peak is contributed by other components than the cell wall as well. Furthermore, the signal of the 730-cm^−1^ peak in Gram-negative bacteria is smaller than that in Gram-positive bacteria, which can be attributed to the following two facts: only Gram-negative bacteria have a thick outer membrane; the thickness of the peptidoglycan layer of Gram-positive bacteria (10 to 20 layers) is larger than that of Gram-negative bacteria (one to three layers). This character also supports that the 730-cm^−1^ peak is dominated by the peptidoglycan layer. In comparison, the 730-cm^−1^ peak is absent in the SERS spectra of mycobacteria (**Fig. 2C**). This distinct behavior may be owing to the existence of long fatty acid chains in the hydrophobic outer membrane of mycobacteria, hindering the peptidoglycan layer from being close to the SERS substrate and thus diminishing the 730 and 1330-cm^−1^ peaks [21]. As a result, the compositions of the outer membrane, consisting of arabinogalactan, mycolic acids, lipids, and pore-forming proteins (MspA), dominate the contribution in the observed vastly complicated SERS spectra. The SERS spectra of the individual compositions are thus needed to identify the molecular origins of the spectra of **Fig. 2C**.

Another novel application of the SERS protocol for live bacteria is its use for assessing a bacteria's susceptibility to an antibiotic. SERS analysis is extremely sensitive and rapid (<1 hr) when used to assess sensitivity to beta-lactam antibiotics that directly target the bacterial cell wall. Interestingly, after the initial SERS perturbation by oxacillin, the Gram-positive *S. aureus* bacteria exhibit a transitory SERS recovery (**Fig. 4A**). We reason that this is because the early effects of the antibiotic as it perturbs the biosynthesis of the cell wall, can be compensated for by the thick layer of murein or peptidoglycan typically found in most Gram-positive bacteria. The Gram-negative bacteria do not possess such a reserve and their response to a beta-lactam antibiotic was found to be progressive and irreversible (**Fig. 4B**). On the other hand, discernible SERS changes in response to antibiotics that interfere with general bacterial proteins synthesis are not evident until after 9∼12 hr of antibiotic treatment. This is because cell wall integrity is able to be maintained for a long time even in the absence of new protein synthesis [22]. Finally, the SERS system established here is capable of assessing the characteristics of a single (live) bacterium and measuring the bacterium's antibiotic sensitivity. This novel platform provides an unprecedented opportunity to study the physiological processes of an individual bacterium. This ought to allow SERS to be used to perform clinical microbial diagnostics directly on a clinical specimen without the need for the time-consuming and sometime hard to carry out creation of a bacterial pure culture.

## Materials and Methods

### Preparation of bacteria samples

*Staphylococcus aureus* (ATCC 25923), *Enterococcus feacali*s (ATCC 29212), *Listeria monocytogen* (ATCC 7644), *Escherichia coli* (ATCC 25922), *Serratia marcescens* (ATCC 8100) and *Klebsiella pneumoniae* (ATCC 13883) were obtained from American Type Culture Collection (ATCC). The bacteria were grown in 5 mL LB broth (Difco) for 14 hr then sub-cultured at OD_600_ = ∼0.5, which was taken as the beginning of the exponential growth phase. They were then washed three times with water and re-suspended in 20 µL of water. *M. tuberculosis* and *M. gordonae* were obtained from Taipei City Hospital and grown in Lowenstein-Jensen medium or 7H9 broth (Difco). For SERS experiments, 3∼5 µL of bacteria suspension was placed on the Ag/AAO substrate that has been treated with oxygen plasma, dried in a laminar-flow cabinet for 5 min, then mounted with 0.5% agarose gel to immobilize the bacterial samples relative to the substrate. Solutions containing the antibiotic of interest were added on top of the agarose and allowed to diffuse towards the bacteria.

### Fabrication of the SERS-active substrate

The SERS-active Ag/AAO substrate consisted arrays of Ag nanoparticles partially embedded in AAO nanochannels; this was manufactured according to the methods described previously [14]. Briefly, the AAO nanochannels substrate was fabricated by anodizing finely polished aluminum foil. The substrate was chemically etched to widen the pore diameter to 25 nm while reducing the channel wall thickness to 5 nm. An electrochemical plating procedure was then employed to grow Ag into the nanaochannels to form an array of nanoparticles with 100 nm in length.

### Raman instrumentation and data processing

Raman spectromicroscopy measurements were performed on a Raman microscope (HR800, Jobin-Yvon) equipped with a HeNe laser at 632.8 nm and NA 0.95 100× water-immersion objective lens. Individual single bacteria or clusters of bacteria could be easily identified under this microscope system. After passing through a narrow band-pass filter to remove residual plasma lines, the laser beam was focused to a ∼1 µm spot on the specimen, which provided a beam intensity of ∼10^5^ W/cm^2^. The scattering radiation was collected by the same objective lens and sent through a Raman notch filter to an 80-cm monochromator. The dispersed spectrum was detected by a LN_2_-cooled charge-coupled device camera. The low laser power density used here prevented adverse effects that might be associated with laser illumination, including local heating, deformation of the Ag-nanoparticles and photo-oxidation. Raman signals were collected from the information-rich part of the spectrum between 400 and 1600 cm^−1^ using an integration time that varied from 1 to 3 sec. The raw SERS readout datasets were processed using algorithms developed in our laboratory to remove noise due to three major sources: a median filter with noise estimation was applied to eliminate any sharp variations caused by cosmic rays, a wavelet de-noising technique was used to smooth out high-frequency noise, and iterative curve fitting to estimate and remove the background baseline due to the noise effect of environmental light. Finally, the spectra were normalized so that the photon count of the highest peak at 732 nm was set to 1. All procedures were performed on a platform that used MATLAB version 7.3.

### Preparations of protoplasts or spheroplasts

To prepare protoplasts, *S. aureus* was cultured overnight and then adjusted to OD_600_ = 0.7 in LB medium. A hypertonic buffer (0.7 M sucrose, 0.02 M maleate and 0.02 M MgC1_2_, pH 6.5) supplemented with lysostaphin (100 µg/ml) and lysozyme (60 µg/mL) was added to the bacteria at 37°C for 15 min with gentle shaking. The extent of cell wall digestion was measured by OD_540_ absorbance. The resulting protoplasts were purified by centrifugation and washed by 0.05 M Tris buffer. To obtain spheroplasts, *E. coli* bacteria were similarly prepared in 10 mM Tris-HCl (pH 7.5) supplemented with 0.75 M sucrose and treated with 2 mg/mL lysozyme in the presence of 1 mM EDTA, and incubated on ice for 10∼20 min. The method converted >99% of the *E. coli* bacteria to spheroplasts.

### Antibiotic treatment

Oxacillin, ampicillin, vancomycin, cefotaxime, gentamicin and tetracycline were purchased from Sigma-Aldrich. The minimal inhibitory concentrations (MIC) for each individual antibiotic with either *S. aureus* or *E. coli* was determined.

### Scanning electron microscopy

The prepared bacteria samples were spotted and dried on a specimen stub. Scanning electron microscopy was done on JEOL JSM-5300 SEM; the accelerating voltage was in the range of 5 to 10 kV.

## References

[1] Mothershed EA, Whitney AM (2006). Nucleic acid-based methods for the detection of bacterial pathogens: present and future considerations for the clinical laboratory.. Clin Chim Acta.

[2] Rolain JM, Mallet MN, Fournier PE, Raoult D (2004). Real-time PCR for universal antibiotic susceptibility testing.. J Antimicrob Chemother.

[3] Kneip K, Moskovits M, Kneip H (2006). Surface-Enhanced Raman Scattering: Physics and Applications Topics in Applied Physics.

[4] Kennedy BJSS, Dickey M, Carron KT (1999). Determination of the distance dependence and experimental effects for modified SERS substrates based on self-assembled monolayers fourmed using alkanethiols.. J Phys Chem B.

[5] Nie S, Emory SR (1997). Probing Single Molecules and Single Nanoparticles by Surface-Enhanced Raman Scattering.. Science.

[6] Efrima S, Bronk BV (1998). Silver colloids impregnating or coating bacteria.. J Phys Chem B.

[7] Jarvis RM, Goodacre R (2004). Discrimination of bacteria using surface-enhanced Raman spectroscopy.. Anal Chem.

[8] Premasiri WR, Moir DT, Klempner MS, Krieger N, Jones G, Ziegler LD (2005). Characterization of the surface enhanced raman scattering (SERS) of bacteria.. J Phys Chem B.

[9] Sengupta A, Laucks ML, Davis EJ (2005). Surface-enhanced Raman spectroscopy of bacteria and pollen.. Appl Spectrosc.

[10] Jarvis RM, Brooker A, Goodacre R (2006). Surface-enhanced Raman scattering for the rapid discrimination of bacteria.. Faraday Discuss.

[11] Naja G, Bouvrette P, Hrapovic S, Luong JH (2007). Raman-based detection of bacteria using silver nanoparticles conjugated with antibodies.. Analyst.

[12] Szeghalmi A, Kaminskyj S, Rosch P, Popp J, Gough KM (2007). Time fluctuations and imaging in the SERS spectra of fungal hypha grown on nanostructured substrates.. J Phys Chem B.

[13] Kahraman M, Yazici MM, Sahin F, Bayrak OF, Culha M (2007). Reproducible surface-enhanced Raman scattering spectra of bacteria on aggregated silver nanoparticles.. Appl Spectrosc.

[14] Wang H-H, Liu C-Y, Wu S-B, Liu N-W, Peng C-Y (2006). Highly Raman-Enhancing Substrates Based on Silver Nanoparticle Arrays with Tunable Sub-10 nm Gaps.. Adv Mater.

[15] Shockman GD, Kolb JJ, Toennies G (1958). Relations between bacterial cell wall synthesis, growth phase, and autolysis.. J Biol Chem.

[16] Glaser L, Lindsay B (1977). Relation between cell wall turnover and cell growth in Bacillus subtilis.. J Bacteriol.

[17] Walker SL, Hill JE, Redman JA, Elimelech M (2005). Influence of growth phase on adhesion kinetics of Escherichia coli D21g.. Appl Environ Microbiol.

[18] Jiang W, Saxena A, Song B, Ward BB, Beveridge TJ (2004). Elucidation of functional groups on gram-positive and gram-negative bacterial surfaces using infrared spectroscopy.. Langmuir.

[19] Premasiri WR, Moir DT, Ziegler LD

[20] Sfrima S, Zeiri L (2008). Understanding SERS of bacteria.. J Raman Spectrosc.

[21] Hoffmann C, Leis A, Niederweis M, Plitzko JM, Engelhardt H (2008). Disclosure of the mycobacterial outer membrane: cryo-electron tomography and vitreous sections reveal the lipid bilayer structure.. Proc Nat Acad Sci.

[22] Franklin TJ, Snow GA (2005). Biochemistry and molecular biology of antimicrobial drug action.

